# *BRCA1* and *ERCC1* mRNA levels are associated with lymph node metastasis in Chinese patients with colorectal cancer

**DOI:** 10.1186/1471-2407-13-103

**Published:** 2013-03-06

**Authors:** Lu Yuanming, Zhang Lineng, Song Baorong, Peng Junjie, Cai Sanjun

**Affiliations:** 1 Department of Colorectal Cancer Center, Fudan University Shanghai Cancer Center, Dong An Road 270, Shanghai, 200032, China; 2 Department of Oncology, Shanghai Medical College Fudan University, Dong An Road 270, Shanghai, 200032, China; 3 Department of Molecular biology and Biochemistry, Shanghai Medical College of Fudan University, Shanghai, 200032, China

**Keywords:** Biomarkers, RT-PCR, Chemosensitivity

## Abstract

**Background:**

Although both excision repair cross-complementing group 1 (ERCC1) and breast cancer susceptibility gene 1 (BRCA1) can be effective biomarkers for chemosensitivity in primary malignant tumors, their applicability to metastases is poorly understood. Here, ERCC1 and BRCA1, which are linked to lymph node metastasis (LNM) in colorectal cancer (CRC), were evaluated in primary CRC samples from Chinese patients with LNM (LNM CRC) or without LNM (non-LNM CRC). mRNA levels of *ERCC1* and *BRCA1* in CRC samples, and their relationships to primary CRC and LNM, were also examined.

**Methods:**

Differences in *BRCA1* and *ERCC1* gene expression between primary CRC with or without LNM were assessed in CRC samples from 120 Chinese patients, using real-time polymerase chain reaction. Relationships between ERCC1 and BRCA1 expression and clinicopathological parameters and prognoses were also examined.

**Results:**

ERCC1 and BRCA1 were significantly down-regulated in LNM CRC compared with non-LNM CRC. Down-expression of ERCC1 and BRCA1 was significantly associated with LNM (*P* < 0.001), advanced TNM stage (*P* < 0.001), and decreased 5-year overall survival rate (*P* < 0.001). Univariate and multivariate analyses showed ERCC1 and BRCA1 expression as independent predictors of recurrence and survival in CRC patients (*P* < 0.05).

**Conclusions:**

*ERCC1* and *BRCA1* mRNA expression levels correlate inversely to CRC metastasis. ERCC1 and BRCA1 might serve as biomarkers for LNM and as prognostic indicators for CRC; their down-expressions are predictors of poor outcome in CRC patients.

## Background

The incidence of colorectal cancer (CRC) is higher in the United States than in China, where it is the third leading cause of cancer-related death in both sexes. However, its rate in China has increased steeply in recent years. Most Chinese patients with CRC have metastatic disease at diagnosis; earlier detection of their disease would greatly improve their odds of survival [1]. Although recent advances in chemotherapy have prolonged survival of patients with advanced disease, these treatments are handicapped by the lack of early-presenting biomarkers for CRC metastasis. Exploration of candidate genes to establish potent biomarkers for earlier detection of lymph node metastasis (LNM) would permit adoption of more suitable chemotherapeutic regimens, although prognoses of patients with CRC are also affected by such factors as tumor localization, quality of surgical procedures, gender, age, and patient’s overall performance status. Monitoring of high-risk individuals increases their 5-year survival rate and decreases chances of cumulative recurrence.

The excision repair cross-complementing group 1 gene (*ERCC1*) is an essential member of the nucleotide excision repair (NER) pathway, which accounts for most platinum–DNA adduct repairs. ERCC1 has been established as a useful molecular marker for NER activity. Early studies have shown that higher *ERCC1* mRNA levels are associated with more active DNA repair processes in various tissues [2]. Interestingly, ERCC1 expression is also associated with cellular and clinical resistance to platinum compounds and to platinum-based chemotherapy, including those for lung and gastric malignancies [3,4].

Breast cancer susceptibility gene 1 (*BRCA1*) is an essential component of several DNA-repair pathways that affect homologous recombination repair, non-homologous repair and NER. BRCA1 is considered to be a differential modulator of tumor response to cisplatin and taxanes [5-7], and BRCA1 levels are reportedly associated with chemosensitivity to cisplatin [8] and taxanes [9,10]. Although the aforementioned studies suggest that both ERCC1 and BRCA1 are effective biomarkers for chemosensitivity in primary tumors, information on their expression in metastases is limited. Therefore, we explored the applicability of these biomarkers as predictive factors in CRC metastasis.

The current study is thus designed to investigate the possibility of using ERCC1 and BRCA1 as biomarkers in CRC metastatic specimens from Chinese patients. We examined mRNA levels of *ERCC1* and *BRCA1* in CRC with LNM (LNM CRC) or without LNM (non-LNM CRC), using real-time quantitative polymerase chain reaction (RT-PCR). We also verified the relationship of ERCC1 and BRCA1 levels on prognosis in CRC patients.

## Methods

### Patient population and characteristics of tissue samples

Samples from a total of 120 patients with colorectal carcinoma were collected from surgical resections performed in our hospital (Fudan University Shanghai Cancer Center, Shanghai, China), after obtaining informed consent. None of the patients received chemotherapy or radiotherapy before surgery. Resected specimens were reviewed by two senior pathologists according to the criteria described in the American Joint Committee on Cancer’s Cancer Staging Manual (7th edition, 2010) [11]. At least 12 lymph nodes each were retrieved from patients with non-LNM CRC, none of whom had distant metastasis. The fresh colorectal tumor tissues were obtained immediately after surgery, washed twice with chilled phosphate-buffered saline (PBS), immediately stored in liquid nitrogen and at ^–^80°C in our tissue bank until further use. Ethical approval was obtained from the Cancer Center Research Ethics Committee of Fudan University.

### Gene expression analysis by real-time quantitative PCR

*ERCC1* and *BRCA1* gene expression was assessed in SYBR Green Supermix (Promega). Samples were treated using a laser capture microdissection technique (Palm Microlaser, Oberlensheim, Germany) to ensure a minimum of 80% of tumor tissue. RNA was then extracted with phenol-chloroform-isoamyl alcohol, followed by precipitation with isopropanol in the presence of glycogen and sodium acetate, resuspension in diethyl pyrocarbonate water (Ambion Inc., Austin, TX), and treatment with DNAse I (Ambion Inc., Austin, TX) to avoid DNA contamination. Complementary DNA was synthesized using Maloney Murine Leukemia Virus retrotranscriptase enzyme. Template cDNA was added to Taqman Universal Master Mix (AB, Applied Biosystems, Foster City, CA) in a 12.5-μl reaction with specific primers and probe for each gene. Primer and probe sets were designed using Primer Express 2.0 Software (AB) and RefSeq sequences (http://www.ncbi.clm.cih.gob/gene). Quantification of gene expression was carried out using the ABI Prism 7900HT Sequence Detection System (AB).

Relative gene expression quantification was calculated according to the comparative cycle threshold (Ct) method [12] using *β-actin* as an endogenous control and commercial RNA controls (Stratagene, La Jolla, CA) as calibrators. Final results were determined as follows: 2^– (ΔCt sample – ΔCt calibrator)^, where ΔCt values of the calibrator and sample are determined by subtracting the Ct value of the target gene from the value of the *β-actin* gene. In all experiments, only triplicates with a standard deviation of the Ct value < 0.20 were accepted. In addition, for each sample analyzed, a retrotranscriptase minus control was run in the same plate to assure lack of genomic DNA contamination.

### Western blotting

Briefly, 30-μg protein samples from each case were separated by 10% sodium dodecyl sulfate polyacrylamide gel electrophoresis and subsequently transferred to poly (vinylidene fluoride) membranes. The membranes were incubated with rabbit polyclonal antibody against ERCC1 or BRCA1 (1:1000 dilution; Abcam, Cambridge, UK) and then incubated with a horseradish-peroxidase-conjugated secondary antibody (1:100 dilution; Proteintech, Chicago, IL, USA). β-Actin was detected simultaneously as a loading control (anti-β-actin, 1:1000 dilution; Kangchen, Beijing, China). All blots were visualized using an ECL detection system (Amersham, Arlington Heights, IL, USA) and quantitated by densitometry using an LAS-3000 imager.

### Immunohistochemistry

Both ERCC1 and BRCA1 expression were examined immunohistochemically using paraffin-embedded tissues. In brief, 4-μm-thick tissue sections were heated in 6.5 mmol/L citrate buffer (pH 6.0) at 100°C for 28 min, and incubated with antibodies against ERCC1 or BRCA1 (1:200 dilution). Immunostaining was performed using the DAKO En-Vision System (Dako Diagnostics, Zug, Switzerland). In the negative control group, PBS was used instead of primary antibody. Expression was scored by two independent experienced pathologists. Each sample was graded according to intensity and extent of staining. The intensity of staining was scored as 0 (no staining), 1 (weak staining), and 2 (strong staining). The extent of staining was based on the percentage of positive tumor cells: 0 (no staining), 1 (1–25%), 2 (26–50%), 3 (51–75%), and 4 (76–100%). These two scores were added together for a final score. The case was considered negative if the final score was 0 or 1 (−) or 2 or 3 (±), and positive if the score was 4 or 5 (+) or 6 or 7 (++). In most cases, the two reviewers provided consistent results. Any inconsistencies were resolved by discussion to achieve a consensus score.

### Statistical analysis

The PCR analysis results were expressed as ratios between two absolute measurements (gene of interest/ internal reference gene). Student’s *t* test was used to evaluate differences in ERCC1 and BRCA1 expression between LNM CRC and non-LNM CRC. The χ^2^ test was used to assess relationships between ERCC1 and BRCA1 expression and clinicopathological factors. The cumulative recurrence and survival probability were estimated using the Kaplan–Meier method; differences were calculated by log-rank test. Prognostic factors were determined using Cox regression analysis. Recurrence-free and overall survival times were calculated from the first resection of the primary tumor to first evidence of recurrence or to death from any cause, respectively. The diagnosis of recurrence was based on the typical features presented on computed tomography/magnetic resonance imaging and elevated serum carcinoembryonic antigen. All *P* values were two-sided; *P* < 0.05 was considered to be significant. Statistical analyses used SPSS 13.0 software.

## Results

### Confirmation of ERCC1 and BRCA1 expression in non-LNM and LNM CRC specimens

**Figure 1:**
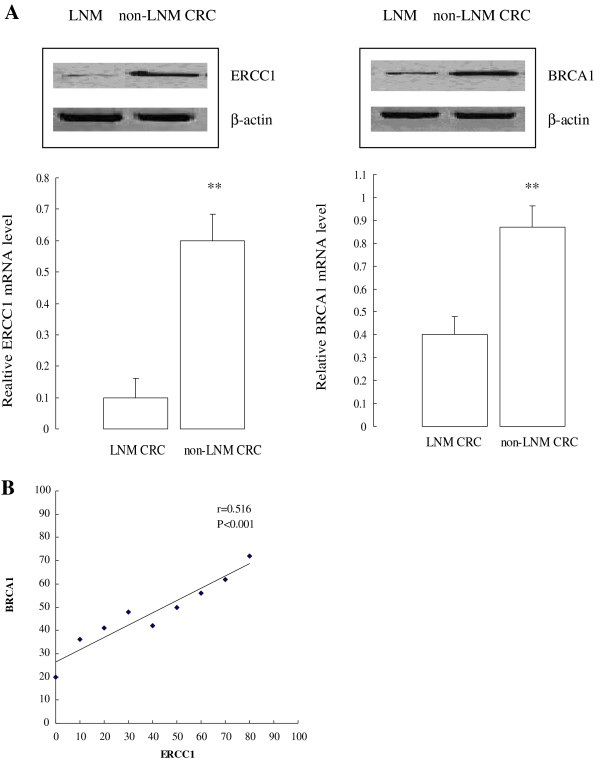
Confirmation of the overexpression of BRCA1 and ERCC1 in colorectal cancer.

Real-time quantitative PCR was used to analyze *ERCC1* and *BRCA1* expression in different groups of CRC. Relative gene expression quantifications were calculated according to the comparative *Ct* method using *β-actin* as an endogenous control. Median *ERCC1* mRNA expression was 6.6 in non-LNM CRC (range: 2.8–8.52; n = 60) and 3.4 in LNM CRC (range: 2.2–8.16; n = 60). Median *BRCA1* mRNA expression was 4.5 in non-LNM CRC (range: 3.21–10.52; n = 60) and 2.6 in LNM CRC (range 1.3–10.16; n = 60). Both *ERCC1* and *BRCA1* mRNA expression were down-regulated in LNM CRC compared with non-LNM CRC. Significant correlations between *ERCC1* and *BRCA1* expression levels (Spearman *r* = 0.516; *P* < 0.001) are shown in Figure 1.

To compare the RT-PCR results with ERCC1 and BRCA1 protein levels, we extended the experiments in the same samples described above with western blot. Thirty micrograms of total proteins from LNM CRC and non-LNM CRC were analyzed using western blotting. Expression of ERCC1 and BRCA1 was dramatically higher in non-LNM CRC compared with LNM CRC (*P* < 0.001). A representative western blotting result is presented in Figure 1A.

### Association of ERCC1 and BRCA1 expression with clinicopathological features and postoperative prognosis of patients with CRC

To study the relationships between ERCC1 or BRCA1 expression and clinicopathological features, and to assess whether ERCC1 or BRCA1 levels could predict clinical outcomes for patients with CRC, an immunohistochemistry study was used to confirm the PCR results, using the same samples.

Statistical analysis showed positive expressions of ERCC1 and BRCA1 were significantly associated with LNM, and advanced TNM stage (*P* < 0.001). However, no significant correlations were observed between ERCC1 or BRCA1 expression and other clinicopathological parameters of sex, age, tumor size, tumor differentiation and tumor location (Tables 1 and 2).

**Table 1:** BRCA1 expression and relationship with clinicopathological factors in CRC

**Clinicopathological factors**	**n**	**BRCA1 expression**		***P *value**^**1**^
		**Negative**	**Positive**	
Sex				
Male	61	27	34	0.205
Female	59	26	33	
Age (yr)				
≤ 60	80	40	40	0.236
> 60	40	16	24	
Tumor size (cm)				
≤ 5	84	40	44	0.134
> 5	36	18	18	
Tumor location				
Colon	45	20	25	0.508
Rectum	75	32	43	
Tumor differentiation ^2^				
I-II	92	48	44	0.132
III-IV	28	10	18	
Tumor status ^2^				
T1-2	42	21	21	0.384
T3-4	78	39	39	
Lymph node metastasis ^2^				
N0	42	22	20	< 0.001
N1-2	78	66	12	
TNM stage ^2^				
I-II	42	22	20	< 0.001
III-IV	78	67	11	

**Table 2:** ERCC1 expression and relationship with clinicopathological factors in CRC

**Clinicopathological factors**	**n**	**ERCC1 expression**		***P *value**^**1**^
		**Negative**	**Positive**	
Sex				
Male	61	29	32	0.325
Female	59	29	30	
Age (yr)				
≤ 60	80	38	42	0.367
> 60	40	20	20	
Tumor size (cm)				
≤ 5	84	42	42	0.157
> 5	36	16	20	
Tumor location				
Colon	45	19	26	0.521
Rectum	75	37	38	
Tumor differentiation ^2^				
I-II	92	52	40	0.124
III-IV	28	12	16	
Tumor status ^2^				
T1-2	42	20	22	0.404
T3-4	78	41	37	
Lymph node metastasis ^2^				
N0	42	18	24	< 0.001
N1-2	78	70	8	
TNM stage ^2^				
I-II	42	21	21	< 0.001
III-IV	78	65	13	

^1^Statistical analysis was estimated with χ^2^ test, and *P* < 0.05 was considered statistically significant;

^2^Grading of differentiation status and TNM classification for colorectal cancer were based on the American Joint Committee on Cancer Cancer Staging Manual (7th edition, 2009). The tumors were classified into two groups: well differentiated (grades I and II) and poorly differentiated (grades III and IV).

**Figure 2:**
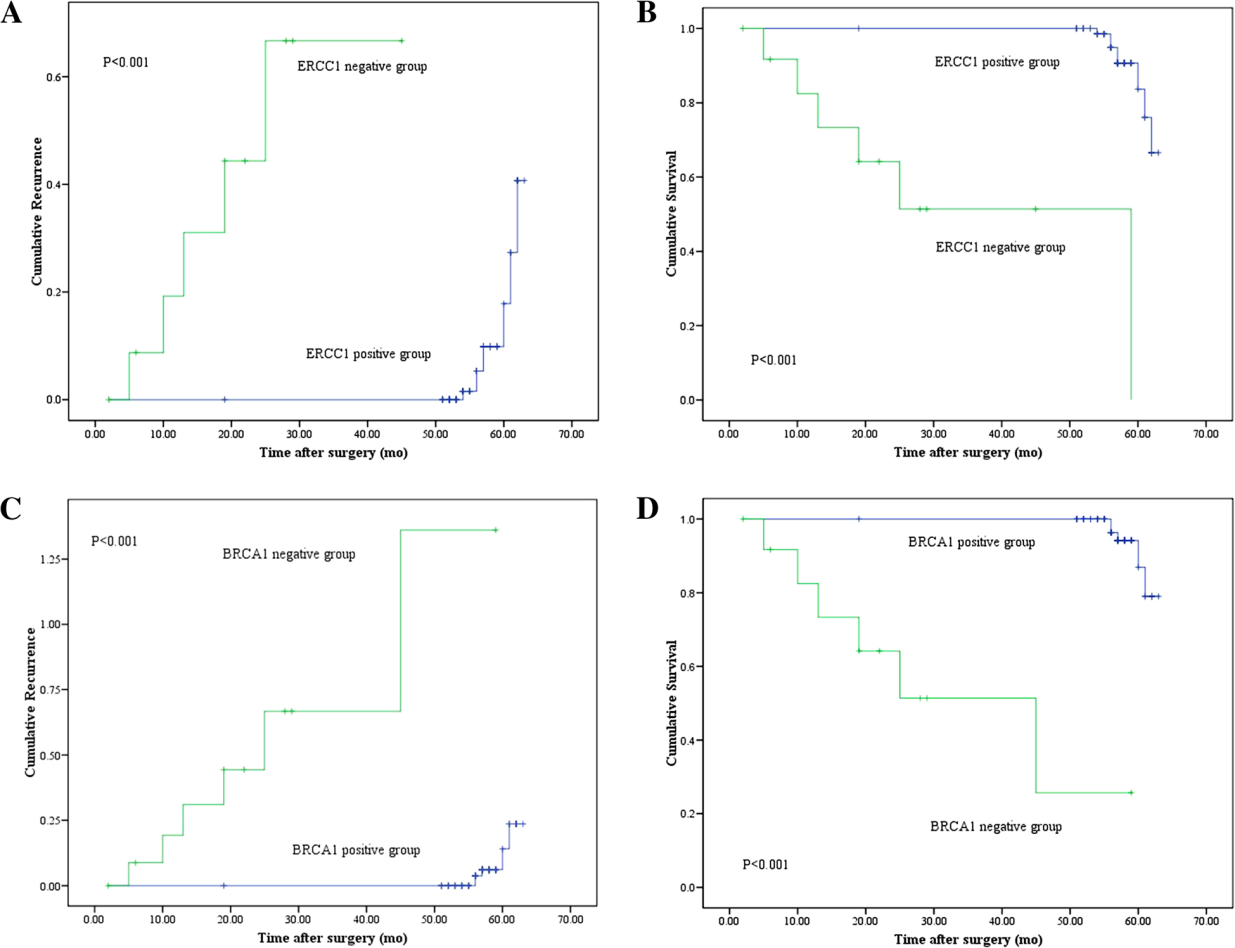
Expression of ERCC1 and BRCA1 correlated with poor prognosis in colorectal cancer patients.

Furthermore, we have found that patients whose CRC specimens were negative for ERCC1 or BRCA1 had significantly poorer prognoses than those with ERCC1^+^/BRCA1^+^ CRC (Figure 2). The 5-year cumulative recurrence rate was significantly higher for patients in the ERCC1^+^/BRCA1^+^ group (*P <* 0.05). The 5-year estimated probability cumulative survival rate was also different in both group patients with BRCA1^–^ CRC or ERCC1^–^ CRC than in the ERCC1^+^/BRCA1^+^ group (*P <* 0.05). Univariate analyses revealed that LNM, TNM stage, ERCC1 expression and BRCA1 expression were related to recurrence and overall survival. In multivariate analysis, LNM, TNM stage, ERCC1 expression and BRCA1 expression were also independent prognostic factors for recurrence and overall survival (*P <* 0.05, Table 3).

**Table 3:** Univariate and multivariate analyses of recurrence and survival (Cox regression)

**Variables**	**Recurrence**		**Survival**	
	**HR (95% CI)**	***P *value**	**HR (95% CI)**	***P *value**
Univariate analysis				
Sex				
Male/female	0.813 (0.479-1.518)	0.604	0.829 (0.424-1.618)	0.662
Age (yr)				
≤ 60/> 60	1.506 (0.804-2.712)	0.163	1.822 (0.947-3.528)	0.065
Tumor size (cm)				
≤ 5/> 5	0.876 (0.163-0.665)	0.687	0.880 (0.438-1.723)	0.704
Tumor location				
Colon/rectum	0.812 (0.445-1.423)	0.518	0.904 (0.476-1.734)	0.778
Tumor differentiation				
I-II/III-IV	1.212 (0.654-2.308)	0.501	1.151 (0.576-2.358)	0.650
Tumor status				
T1-2/T3-4	0.866 (0.475-1.618)	0.687	1.020 (0.504-2.028)	0.904
Lymph node metastasis				
N0/N1-2	2.707 (1.502-4.912)	0.011	2.812 (1.413-5.509)	0.012
TNM stage				
I-II/III-IV	3.554 (1.932-6.526)	<0.001	3.385 (1.677-6.843)	<0.001
ERCC1 expression				
Negative/positive	3.556 (1.919-6.932)	<0.001	4.038 (1.913-8.712)	<0.001
BRCA1 expression				
Negative/positive	3.244 (1.191-7.012)	<0.001	4.033 (1.906-8.512)	<0.001
Multivariate analysis				
LNM				
N0/N1-2	0.210 (0.051-0.758)	0.018	0.196 (0.041-0.852)	0.028
TNM stage				
I-II/III-IV	8.905 (2.072-38.190)	0.003	9.037 (1.703-48.105)	0.010
ERCC1 expression				
Negative/positive	2.673 (1.134-6.011)	0.018	3.011 (1.236-7.95)	0.031
BRCA1 expression				
Negative/positive	2.401 (1.115-5.722)	0.013	2.844 (1.106-7.44)	0.021

HR: Hazard ratio; CI: Confidence interval.

## Discussion

CRC is an aggressive cancer, with 300,000 newly diagnosed cases and 200,000 CRC-caused deaths each year in Europe and the United States [13]. Biomarkers that could help diagnose CRCs before metastases occur can lead to earlier, more successful treatments. Moreover, accurate biomarkers for metastases could aid clinicians in identifying the most appropriate chemotherapies for patients with CRC who have had resections; up to 50% of patients who undergo potentially curative surgeries ultimately suffer recurrence and die of metastatic disease [14].

More than 85% of CRCs have been attributed to environmental factors, which can produce adducts, damage and strand breaks in DNA. Damaged DNA can be removed and recovered by DNA-repairing enzymes, which are critical for the genome protection and cancer prevention [15,16]; the relationship between DNA repair genes and various cancers has been widely studied [17]. Adjuvant therapy is important to the clinical outcomes of the stage II–III cases that represent approximately 70% of CRC patients; adjuvant treatment following primary surgery could be improved by prognostic biomarkers. In this study, we investigated whether ERCC1 and BRCA1 could be such biomarkers and found that their down-expression is associated with poor prognosis in CRC.

ERCC1 is an endonuclease that helps perform NER of DNA [18]. We found negative expression of ERCC1 to correlate with LNM and advanced TNM stage, implying that ERCC1 decreases CRC metastasis, and by extension, that its reduced expression might be an early event in colorectal carcinogenesis. Patients with CRC that expresses negative ERCC1 have been shown to have high cumulative recurrence and low cumulative survival. On the other hand, the NER pathway is thought to repair DNA damage cause by platinum agents; several studies demonstrated an inverse relationship between impaired DNA-repair capacity and increased response rates to platinum drugs. Patients with CRC whose tumors show low levels of ERCC1 gene amplification reportedly have superior overall survival if treated with fluorouracil/oxaliplatin. However, many factors affect metastasis. ERCC1 could be restrained or promoted by other genes, which could influence LNM somehow; this area is already the subject of advanced research.

BRCA1 also has a role in DNA repair similar to that of ERCC1. Variants of *BRCA1* are markers for breast and ovarian cancers, but it is unclear whether mutations in this gene increase the risk of CRC. Our data show that BRCA1 might be involved in CRC metastasis, as with ERCC1, and therefore it is a potential biomarker for CRC. However, changes in BRCA1 expression seem to be less sensitive to early-stage disease, compared with ERCC1, although changes in expressions of both proteins appear to correlate with LNM. This correlation between ERCC1 or BRCA1 expression and CRC metastasis has been suggested recently [19,20], but no statistical association could be established between reduced expression of ERCC1 or BRCA1 and tumor stage and lymph node involvement. Our data show relationships between negative ERCC1 or BRCA1 expression and clinical CRC LNM, implying that both ERCC1 and BRCA1 are involved in CRC metastasis, and that reduced expression of these proteins are early events in colorectal carcinogenesis.

Arguably, the most important relationship we found was that between negative ERCC1 or BRCA1 expression in CRC and poor patient survival. We found negative ERCC1 or BRCA1 expression to correlate with LNM and advanced TNM stage, which suggests that ERCC1 and/or BRCA1 affect CRC progression from localized to LNM disease. In addition, patients with negative ERCC1 or BRCA1 expression in CRC have increased risk of recurrence and significantly reduced overall survival rates. Univariate and multivariate analyses indicate that ERCC1 or BRCA1 expression could serve as independent prognostic factors for recurrence and overall survival in patients with CRC.

## Conclusions

Our quantitative analysis of mRNA and protein expression showed ERCC1 and BRCA1 to be significantly negatively expressed in LNM CRC. Further evaluation using the same sample set suggests that ERCC1 and BRCA1 are biomarkers for LNM and predictors of prognosis in CRC.

## Abbreviations

BRCA1: Breast cancer susceptibility gene 1; CRC: Colorectal cancer; ERCC1: Excision repair cross-complementing group 1; LMN: Lymph node metastasis; LMN CRC: Colorectal cancer with lymph node metastasis; non-LNM CRC: Colorectal cancer without lymph node metastasis; PBS: Phosphate-buffered saline; RT-PCR: Real-time quantitative polymerase chain reaction

## Competing interests

The authors declare that they have no competing interests.

## Authors’ contributions

Lu Yuanming designed the study and took part in the experiments. Zhang Lineng and Song Baorong performed most of the experiments. Peng Junjie was responsible for sample collection. Cai Sanjun directed the study. All authors read and approved the final manuscript.

## Pre-publication history

The pre-publication history for this paper can be accessed here:

http://www.biomedcentral.com/1471-2407/13/103/prepub
